# Candidate high myopia loci on chromosomes 18p and 12q do not play a major role in susceptibility to common myopia

**DOI:** 10.1186/1471-2350-5-20

**Published:** 2004-08-03

**Authors:** Grace Ibay, Betty Doan, Lauren Reider, Debra Dana, Melissa Schlifka, Heping Hu, Taura Holmes, Jennifer O'Neill, Robert Owens, Elise Ciner, Joan E Bailey–Wilson, Dwight Stambolian

**Affiliations:** 1 Inherited Disease Research Branch, National Human Genome Research Institute, National Institutes of Health, 333 Cassell Dr., Suite 2000, Baltimore, MD 21224, USA; 2 Dept. of Ophthalmology, University of Pennsylvania, 3535 Market St., Suite 701, Philadelphia, PA 19104, USA; 3 Owens Optometrics, 654 E. Main St., New Holland, PA 17557, USA; 4 Pennsylvania College of Optometry, 8360 Old York Rd., Elkins Park, PA 19027, USA

## Abstract

**Background:**

To determine whether previously reported loci predisposing to nonsyndromic high myopia show linkage to common myopia in pedigrees from two ethnic groups: Ashkenazi Jewish and Amish. We hypothesized that these high myopia loci might exhibit allelic heterogeneity and be responsible for moderate /mild or common myopia.

**Methods:**

Cycloplegic and manifest refraction were performed on 38 Jewish and 40 Amish families. Individuals with at least -1.00 D in each meridian of both eyes were classified as myopic. Genomic DNA was genotyped with 12 markers on chromosomes 12q21-23 and 18p11.3. Parametric and nonparametric linkage analyses were conducted to determine whether susceptibility alleles at these loci are important in families with less severe, clinical forms of myopia.

**Results:**

There was no strong evidence of linkage of common myopia to these candidate regions: all two-point and multipoint heterogeneity LOD scores were < 1.0 and non-parametric linkage p-values were > 0.01. However, one Amish family showed slight evidence of linkage (LOD>1.0) on 12q; another 3 Amish families each gave LOD >1.0 on 18p; and 3 Jewish families each gave LOD >1.0 on 12q.

**Conclusions:**

Significant evidence of linkage (LOD> 3) of myopia was not found on chromosome 18p or 12q loci in these families. These results suggest that these loci do not play a major role in the causation of common myopia in our families studied.

## Background

Myopia is one of the leading causes of vision loss around the world[1]. In the United States, myopia affects approximately 25% of adult Americans[2]. Ethnic diversity appears to distinguish different groups with regard to prevalence. Caucasians have a higher prevalence than African Americans[3]. Asian populations have the highest prevalence rates with reports ranging from 50–90%[1,4,5]. Jewish Caucasians, one of the target populations of the present study, have consistently demonstrated a higher myopia prevalence than the general Caucasian population in both U.S. and European population surveys; Orthodox Jewish males in particular show increased susceptibility[6,7].

Despite many decades of research, little is known about the precise molecular defects and abnormal biochemical pathways that result in myopia. Compelling data from familial aggregation and twin studies indicate that susceptibility to myopia is inherited. Several familial aggregation studies have reported a greater prevalence of myopia in children of myopic parents compared to children of nonmyopic parents [8-12]. Several twin studies have demonstrated a very high heritability (estimates ranging from 60 to 90%) for myopia [13-15]. Other recent genetic studies of families with -6.00 D or more of myopia (termed high or pathological myopia) have reported significant linkage to regions on chromosome 18p11.31, 12q21-23, 17q21-22 and 7q36 [16-19]. The 18p candidate region has been confirmed in an independent study of high myopia [20]. Mutti et al.[21] examined the hypothesis that families with milder, juvenile onset myopia might show linkage to these same candidate regions. They found no evidence to support such a role in this more common form of myopia but their study was not highly powered in the presence of heterogeneity. Evidence also exists that myopia may be under environmental influences. The rapid increase in the prevalence of myopia over the last several decades suggests that environmental factors are important [22,23]. Furthermore, studies have shown a positive correlation of specific environmental factors, such as nearwork, with myopia [24,25]. It has been postulated that myopia develops in a person who engages in significant periods of sustained nearwork as an adaptive response to achieve better focus for near images[26]. Interestingly, Cordain et al.[27] suggest a positive correlation for myopia with increased consumption of carbohydrates, hyperinsulinemia and type II diabetes. Finally, experimental findings from animal studies show that the refractive state of young chicks will adapt to compensate for refractive errors induced by spectacle lenses[28].

The combination of genetic and environmental influences on the development of myopia suggests that myopia is a complex disorder and should not be classified as a simple Mendelian trait. Further evidence is shown by studies that have reported correlation coefficients for myopia between offspring and parents and between pairs of siblings to lie between 0.07–0.36 [29-32]. Due to the possible complexity of myopia, population isolates offer many advantages for genome-wide mapping studies[33]. First, they have reduced genetic complexity. Second, the people in most isolates share a common environment and culture. Differences in diet, exercise, sanitary conditions, and exposure to infectious diseases are minimized. A common language and religion usually promote social cohesion. Therefore, some of the environmental noise surrounding complex diseases that are determined by a combination of nature and nurture may be avoided.

To avoid some of the complexity in mapping genes for myopia we have collected refractive measurements and DNA samples from Amish and orthodox Jewish families with myopia. The Old Order Amish are mostly rural farmers and craftsmen. They lead a culturally and technologically distinct lifestyle. They are a genetically well-defined founder population with large families and well-documented genealogies [34,35]. Family history records of the Amish in Lancaster County, Pennsylvania, beginning from 1727 are highly preserved[36]. Other features of this population include a relatively high standard of living, low migratory tendencies, and no practice of birth control, which facilitate the recruitment of large and extended families.

The orthodox Jewish families in this study are all of Ashkenazi descent, a population with known founder effects in other common diseases[37]. This population also has somewhat larger family sizes than average in the US. In this initial report, we describe the design of our study and show that two regions (18p and 12q) previously reported to be linked to high myopia cannot explain the familial aggregation in these families with mostly moderate to milder forms of myopia. We had hypothesized that allelic heterogeneity might exist at these candidate loci such that in addition to highly penetrant alleles for extreme high myopia, there might also exist other susceptibility alleles of (possibly) lower penetrance that produce milder phenotypic forms of myopia. However, we found no strong evidence in support of this hypothesis.

## Methods

### Family screening

The study protocol adhered to the tenet of the Declaration of Helsinki and was approved by the University of Pennsylvania and the National Human Genome Research Institute, National Institutes of Health institutional review boards. Informed consent was obtained from the subjects after explanation of the nature and possible consequences of the study. The collection of orthodox Jewish individuals was begun by a mass mailing of 3900 letters to all the known orthodox Jewish families living in Lakewood, New Jersey. Questionnaires were sent with letters explaining the study. If willing to participate, individuals completed and returned questionnaires that included their contact and physician information. Second and third mailings went out to individuals who did not respond – either positively or negatively – to the first mailing. The total number of questionnaires returned was 1,310. All Jewish individuals included in the study were of Ashkenazi heritage. Collection of Amish families was done by an advertisement in an Amish newspaper, referrals from local eye doctors in the Lancaster County community and word of mouth. Criteria for entry into the study included the following: 1) Negative history of systemic or ocular disease which may predispose to myopia, 2) negative history of a premature birth, 3) proband must be affected and must have a family history of myopia in either their parents or children, 4) only one parent (as opposed to both parents) of the proband can be myopic. For the Orthodox Jewish population, an individual's myopic status was obtained from the most recent (within 2 years) measurement of refractive error. If not recent, an individual was given a repeat exam by their local eye doctor or one of the study investigators (D.S.). For the Amish subjects, all participants were examined by a study investigator (D.S.) at the Amish Eye Clinic in Strasburg, PA. Amish participants were brought to the study site because they do not have phone access making it difficult to obtain a past history and records. Cycloplegic refractions were done on all individuals less than 40 years of age with one drop each of 1% cyclogyl, 1% mydriacyl and 2.5% phenylephrine. A manifest refraction was performed if an individual was older than 40 years of age. Classification as myopic required at least -1.00D in each meridian of both eyes. Individuals were classified as nonmyopes if they were over 21 years of age and did not meet the above criteria for myopia. Other individuals were classified as nonmyopic if they were 5–10 years old and had ≥ +3.00D in each meridian, 10–18 years old with ≥ +2.00D in each meridian or 18–21 years old with ≥ +0.50D in each meridian. All other individuals were classified as unknown for the trait.

This ascertainment protocol resulted in the collection of 40 Amish families and 38 orthodox Jewish families. Of the 40 Amish families, phenotype data were available on 340 persons (170 individuals were affected and 170 were unaffected) but only 323 DNA samples were available to be genotyped. In the 38 Jewish families, phenotype data were available for 313 persons (177 affected, 122 unaffected and 14 of indeterminate phenotype) and DNA samples were available and genotyped for 290 of these family members.

### DNA extraction and genotyping

Peripheral blood was collected from family members. High molecular weight genomic DNA extraction from the blood samples was performed with a kit (Puregene; Gentra Systems, Inc.; Minneapolis, MN, USA). Polymerase chain reactions were performed in a 17.05 ul volume containing 12–320 ng/ul of DNA; 880 uM each of dATP, dCTP, dGTP, and dTTP; 3 mM MgCl_2_; 10 mM Tris/HCl (pH8.3); 50 mM KCl; 0.6 uM of each primer; and 7.6 units/ul of Taq polymerase. Standard thermocycling was as follows: 94°C for 30 sec., 55°C for 30 sec. and 72°C extension time for 30 sec. Markers used included D12S85, D12S1706, D12S346, D12S78, D12S79, D12S86, D18S59, D18S481, D18S63, D18S452, D18S53, and D18S474 located in the 18p and 12q regions implicated in high myopia [16,17].

### Power studies

A simulation study was conducted on the first 44 Ashkenazi Jewish families collected in this study, using the computer program SIMLINK [38,39], to compare the projected power from alternative parametric trait models (five of these families contributed no information about linkage and so were not genotyped and the sixth family was dropped after genotyping because of sample problems that resulted in inadequate linkage information). It was assumed that the myopia trait is controlled by an autosomal dominant bi-allelic locus and the frequency of the high risk allele was varied in different simulations, using both 0.05 and 0.01. The actual observed pedigree structures, trait phenotypes and DNA sample availability were used to simulate the trait locus genotypes and linked and unlinked marker loci were also simulated. A highly polymorphic marker locus (9 equally frequent alleles) was assumed. The power available from these families to detect linkage was evaluated using different models for penetrance and sporadic rates. For each of the 12 models tested, simulations were performed assuming that the underlying proportion of families linked to the same marker locus (α) was 25%, 50%, 75% and 100%. Furthermore, for each model at each specified level of α, simulations were performed for six recombination distances (θ) between the disease and the marker loci (i.e., θ = 0.01, 0.05, 0.1, 0.2, and 0.5); for three maximum penetrances (0.6, 0.7 and 0.8) for gene carriers; and for two phenocopy rates (0.08 and 0.15). LOD scores assuming homogeneity were calculated for each of 100 replicates. The average LOD score over all replicates (ELod) and its standard deviation were calculated for each model simulated. The power of these families to detect a linkage (i.e., to obtain a LOD score ≥ 3.0) was tabulated for the linked marker and the probability of obtaining a LOD score greater than 1.0 when no linkage exists (Type I error) was tabulated for the unlinked marker in all simulations.

### Linkage analysis

The data on 40 Amish and 38 Ashkenazi Jewish families were checked for misspecification of family structures, data entry errors and genotyping errors using the program SIBPAIR[40]. This program was also used to estimate allele frequencies at marker loci from the unrelated founder individuals in the families. Parametric two-point linkage analysis was performed with the MLINK program of the FASTLINK package [41,42] and the utility programs MAKEPED, Linkage Control Program, and Linkage Report Program from LINKAGE 5.1 [43-45]. Intermarker distances (Kosambi cM) of the microsatellite markers were obtained from the Marshfield database : D12S85-42.78-D12S1706-0.53-D12S346-7.22-D12S78-13.44-D12S79-9.23-D12S86; D18S59-6.94-D18S481-1.36-D18S63-10.4-D18S452-22.54-D18S53-30.08-D18S474. To carefully explore the possibility of linkage of common myopia to these high myopia candidate regions, we utilized 12 different parametric models (Table 1). Analyses were performed assuming all combinations of three different frequencies for the myopia susceptibility allele (0.0133, 0.5 and 0.10) and four different sets of genotypic penetrances for the gene carriers and non-gene carriers, respectively: 0.90 and 0.0; 0.80 and 0.0; 0.80 and 0.05; and 0.60 and 0.15. Models 1–4 (Table 1) assume an allele frequency for the putative myopia susceptibility allele of 0.0133, which is the same value used by Young et al. in their linkage studies of high myopia [16,17] and close to the value of 0.01 that showed good power in our power simulation (note that a more frequent allele frequency of 0.05 resulted in similar but always lower predicted power in our simulations than the power obtained when an allele frequency value of 0.01 was used; note also that this allele frequency applies only to the linked trait locus, so that if there are multiple loci and environmental factors involved in causing myopia under a heterogeneity model, any single locus might only account for a small proportion of all myopia cases). No sex difference was assumed in any of these models. All persons younger than age 5 were coded as unknown for the trait. This analysis assumed autosomal dominant inheritance of a myopia susceptibility allele. Recombination fractions were assumed to be equal in men and women. The program HOMOG[46] was used to test for evidence of heterogeneity in the presence of linkage in the two-point parametric linkage analyses. The heterogeneity testing was performed separately in the Jewish and Amish families and also in a joint analysis of LOD scores from the two datasets combined. Multipoint parametric and nonparametric linkage analyses were performed with the GENEHUNTER program[47]. Because of program memory constraints, one large Amish pedigree was split into three small ones for the GENEHUNTER analysis. The parametric analyses in GENEHUNTER used the same models described above, while allowing for locus heterogeneity. The nonparametric statistic NPL_all_, which estimates the statistical significance of alleles shared IBD between all affected family members, was calculated also, together with an estimated *P *value for the Amish and Jewish datasets separately. A nonparametric analysis combining the Amish and Jewish families was then performed by calculating the sum of NPL scores for each family (obtained in the separate Amish and Jewish analyses just described) divided by the square root of the total number of families (N = 78)[48] to obtain an overall combined NPL score.

**Table 1:** Different parametric models utilized for the linkage analysis

Model	Allele Frequency	Penetrance in DD:Dd susceptibility allele carriers	Penetrance in dd normal homozygotes
1	0.0133	0.90	0.00
2	0.0133	0.80	0.00
3	0.0133	0.80	0.05
4	0.0133	0.60	0.15
5	0.05	0.90	0.00
6	0.05	0.80	0.00
7	0.05	0.80	0.05
8	0.05	0.60	0.15
9	0.10	0.90	0.00
10	0.10	0.80	0.00
11	0.10	0.80	0.05
12	0.10	0.60	0.15

## Results

### Power simulation

As expected, the estimated average maximum LOD score decreases with the distance between the linked marker locus and the trait locus, and with increasing heterogeneity. However, only minimal changes in projected power for our Ashkenazi families were observed as penetrance, phenocopy rate and disease allele were varied. Projected power was always higher when an allele frequency of 0.01 was used for the susceptibility allele at the trait locus than when an allele frequency of 0.05 was used; however, these differences in power were very small. Table 2 shows a representative sample of the predicted power results for detecting linkage to a marker 5 cM from the trait locus (the average maximum distance that a trait locus would be from our genotyped markers if it fell within the confines of either of these two candidate regions on 18p and 12q) assuming an autosomal dominant susceptibility allele frequency of 0.01. If all families were linked to one locus, these families were predicted to have 100% power to detect linkage to a marker 5 cM away from the trait locus with a LOD of 3 or more (the ELods were all ≥ 14). As less families were linked to the marker locus (i.e., as genetic heterogeneity increased) the power decreased but was still good (≥ 67%) if 50% or more of the families were linked. Even when only 25% of families were linked to the same locus, the expected LOD score was over 1.0 for all models. Of course, these LOD scores were calculated assuming homogeneity, and it is well known that power can be substantially increased when heterogeneity exists if LOD scores are calculated assuming heterogeneity (HLODs) as we have done in this study. So we would expect our actual power to detect linkage to be even higher than our simulations of heterogeneity predict. Observed Type I error rates were compatible with the nominal Type I error levels for all models. Between 0 and 1% of replicates produced a LOD score > 1 at any test map distance for unlinked markers.

**Table 2:** Power and ELods from 100 replicates of simulated data, dominant susceptibility allele frequency of 0.01

PENETRANCE IN DD:Dd SUSC. ALLELE CARRIERS	PENETRANCE IN dd NORMAL HOMOZYGOTE	% FAMILIES LINKED							
		
		100%		75%		50%		25%	
		Power^1^	ELod ± s.d.^2^	Power^1^	ELod ± s.d.^2^	Power^1^	ELod ± s.d.^2^	Power^1^	ELod ± s.d.^2^
0.8	0.08	100	14.0 ± 0.3	99	8.9 ± 0.3	82	4.7 ± 0.2	18	1.7 ± 0.1
0.8	0.15	100	14.7 ± 0.3	100	9.1 ± 0.3	73	4.8 ± 0.2	14	1.6 ± 0.1
0.7	0.08	100	14.8 ± 0.3	99	8.5 ± 0.3	77	4.8 ± 0.2	17	1.8 ± 0.15
0.7	0.15	100	15.2 ± 0.3	99	8.8 ± 0.3	70	4.5 ± 0.2	14	1.6 ± 0.1
0.6	0.08	100	15.0 ± 0.3	98	8.6 ± 0.25	67	4.3 ± 0.2	14	1.7 ± 0.1
0.6	0.15	100	14.2 ± 0.3	100	8.4 ± 0.3	73	4.3 ± 0.2	5	1.2 ± 0.1

^1^Power = 100 X Proportion of replicate samples that yielded a homogeneity LOD score ≥ 3.0 ^2^ELod = average homogeneity LOD score over all replicates ± its standard deviation

### Linkage

Parametric and nonparametric LOD scores were calculated for 40 Amish pedigrees and 38 Jewish pedigrees. The six markers on chromosome 12q21-q23 spanned 73 cM, and the six markers on chromosome 18p11.2-p11.32 spanned 70 cM. Markers D12S1706, D12S346, D18S59, D18S481 and D18S63 were previously reported by Young et al. [16,17] as showing evidence of linkage to autosomal dominant high myopia. Under all 12 parametric models, the evidence in favor of linkage to these candidate regions was minimal and this evidence varied only slightly as the assumptions of the trait model were changed across the models. Therefore, only the results from model 1 are presented here.

### Two-point linkage analyses in the Amish and Jewish populations

Results of two-point parametric linkage analysis of myopia assuming linkage heterogeneity to the chromosome 12 and 18 markers in 40 Amish families are presented in Table 3. Statistically significant or suggestive linkage under locus homogeneity was not observed for either chromosome 12 or chromosome 18. Only one marker, D18S474 showed a two-point LOD ≥ 1.0 (LOD = 1.39 at θ = 0.3). Testing for linkage heterogeneity using HLODs in HOMOG did not significantly improve the evidence for linkage to any of these markers.

**Table 3:** Two-point parametric LOD scores for myopia (Model 1) in 40 Amish Families

	RECOMBINATION FRACTION, θ						
	
MARKER	0.0	0.01	0.05	0.1	0.2	0.3	0.4
D12S85	-19.55	-12.97	-9.02	-6.27	-2.92	-1.15	-0.31
D12S1706	-39.91	-29.33	-17.82	-10.79	-3.85	-1.06	-0.18
D12S346	-36.18	-24.54	-13.68	-7.57	-1.99	-0.14	0.08
D12S78	-40.39	-28.61	-17.98	-11.46	-4.57	-1.5	-0.33
D12S79	-49.21	-32.42	-19.78	-12.36	-4.82	-1.5	-0.2
D12S86	-40.95	-32.39	-20.01	-12.52	-5.14	-1.85	-0.43
D18S59	-26.98	-20.07	-11.74	-6.77	-2.2	-0.48	-0.02
D18S481	-29.61	-19.93	-11.02	-5.99	-1.42	-0.04	0.16
D18S63	-32.32	-23.1	-12.11	-5.98	-0.87	0.5	0.35
D18S452	-38.37	-27.85	-16.16	-9.31	-2.76	-0.35	0.15
D18S53	-35.69	-25.42	-15.08	-9.07	-3.22	-0.77	-0.01
D18S474	-26.64	-14.04	-5.89	-2.01	0.94	1.39	0.7

The same markers on chromosome 12q21-q23 and chromosome 18p11.2-p11.32 were analyzed using 38 Ashkenazi Jewish families (Table 4). Statistically significant or suggestive linkage was not observed on either chromosome, no homogeneity LOD scores ≥ 1.0 were observed, and testing for linkage in the presence of heterogeneity (HLODs in HOMOG) did not alter this result.

**Table 4:** Two-point parametric LOD scores for myopia (Model 1) in 38 Ashkenazi Jewish families

	RECOMBINATION FRACTION, θ						
	
MARKER	0	0.01	0.05	0.1	0.2	0.3	0.4
D12S85	-7.5	-6.71	-4.62	-2.56	-0.44	0.19	0.17
D12S1706	-36.21	-28.66	-17.67	-10.77	-3.85	-1.04	-0.15
D12S346	-32.76	-24.46	-13.34	-7.11	-1.68	-0.04	0.11
D12S78	-27.99	-20.59	-10.1	-4.65	-0.46	0.43	0.24
D12S79	-38.53	-30.26	-18.47	-11.21	-4.12	-1.24	-0.26
D12S86	-39.59	-31.68	-19.83	-12.58	-5.12	-1.8	-0.45
D18S59	-35.34	-27.25	-16.82	-10.69	-4.4	-1.51	-0.26
D18S481	-32.69	-24	-14.58	-9.19	-3.73	-1.26	-0.24
D18S63	-36.39	-28.55	-17.92	-11.36	-4.65	-1.63	-0.35
D18S452	-34.97	-25.21	-15.47	-9.72	-3.78	-1.22	-0.23
D18S53	-38.03	-26.36	-14.65	-8.62	-3.15	-0.94	-0.14
D18S474	-29.88	-22.79	-14.41	-9.29	-3.98	-1.45	-0.31

Heterogeneity testing using HOMOG in the combined Jewish and Amish families also did not yield any significant evidence of linkage in these two regions, with the maximum HLOD's being 0.39 and 0.95 on chromosomes 12 and 18 respectively.

Furthermore, nonparametric two-point NPL scores did not show any significant evidence for linkage in either the Amish or Jewish populations. The observed combined NPL score of 1.37 for D12S1706 approached nominal significance at *p *= 0.09 but was not close to the significance level of at least p = 0.01 needed to provide confirmation of a prior linkage[49].

### Multipoint linkage analyses in the Amish and Jewish populations

Multipoint parametric linkage analyses assuming homogeneity were consistently negative in both the Amish and Jewish datasets. A maximum multipoint parametric HLOD of 0.92 was observed at D18S474 in the Amish population. However, multipoint parametric HLOD scores were essentially zero for the chromosome 12 region in the Amish and for both the chromosome 12 and 18 regions in the Jewish families.

**Figure 1:**
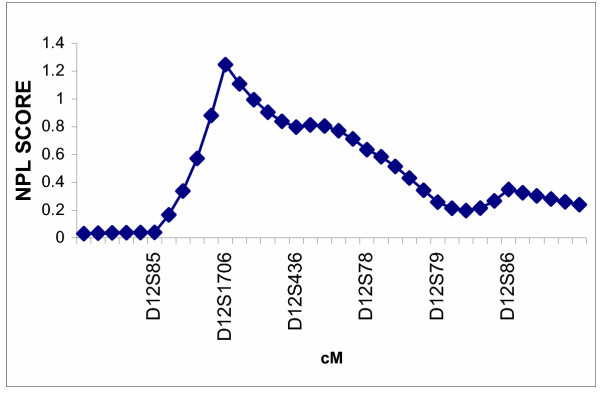
Multipoint nonparametric linkage analysis of myopia to chromosome 12q in 40 Amish families

**Figure 2:**
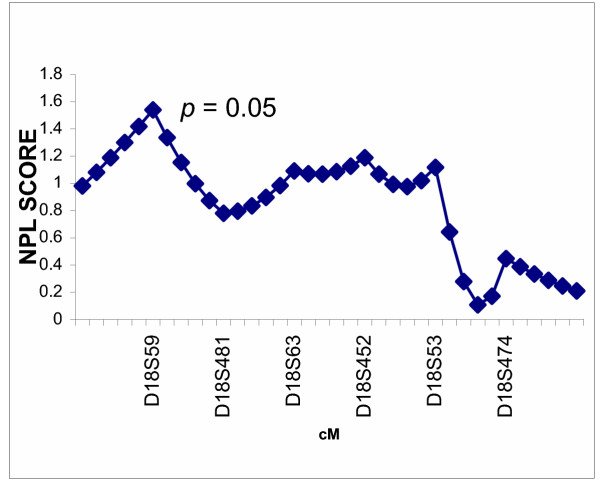
Multipoint nonparametric linkage analysis of myopia to chromosome 18p in 40 Amish families

**Figure 3:**
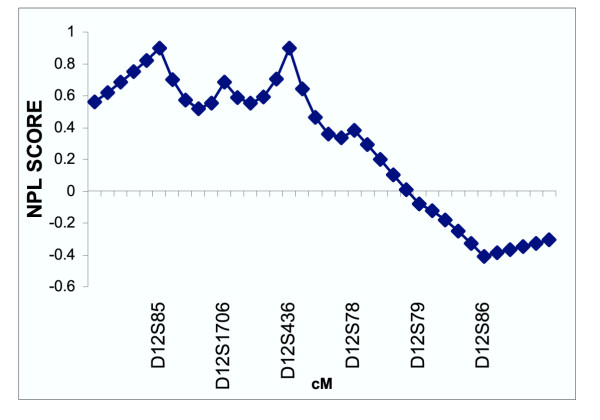
Multipoint nonparametric linkage analysis of myopia to chromosome 12q in 38 Ashkenazi Jewish families

**Figure 4:**
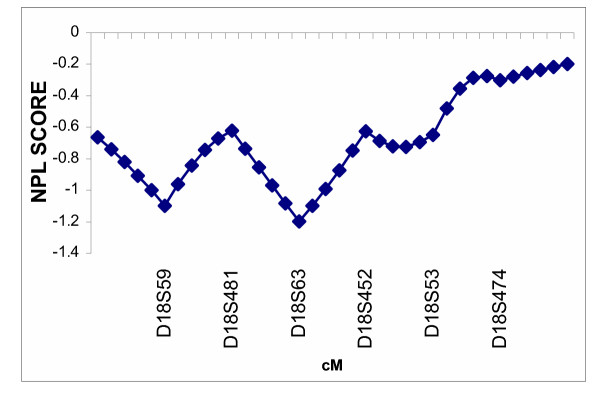
Multipoint nonparametric linkage analysis of myopia to chromosome 18p in 38 Ashkenazi Jewish families

The multipoint nonparametric analyses did not show statistically significant evidence for linkage of myopia to either candidate region in the Amish (Figures 1 and 2) or the Jewish (Figures 3 and 4) families. Only very mild evidence for linkage of myopia in the Amish was observed between markers D18S59 and D18S481 (NPL= 1.54, *p *= 0.05) (Figure 2).

### Individual families showing linkage

Only one Amish family (3061) showed marginal evidence for linkage (LOD > 1.0) to the region previously reported on chromosome 12 (D12S1706 and D12S346) for both two-point and multipoint parametric analyses (Table 5). Three Amish families gave LOD >1.0 at 2 markers on chromosome 18p. In both two-point and multipoint parametric analyses, family 3064 showed mild evidence of linkage (LOD = 1.30) to marker D18S63, and families 3049 and 3053 showed slight evidence of linkage to marker D18S474 (two-point LOD= 1.14 and 1.30, respectively). Simulations using SIMLINK (model 1) showed that for these individual families, the maximum two-point LOD score obtained when a linked marker was simulated at a recombination fraction of 0.0 ranged from 0.99 to 1.4, and the probability of obtaining a LOD over 1.0 for an unlinked marker ranged from <0.01 to 0.036. The nominal significance level corresponding to a LOD score of 1.0 is approximately 0.01. A total of three Jewish families showed marginal evidence for linkage to chromosome 12q markers for both two-point and multipoint analyses, with two-point and multipoint parametric LOD > 1.0. Simulations using SIMLINK (model 1) showed that for these individual families the maximum two-point LOD score obtained when a linked marker was simulated at a recombination fraction of 0.0 ranged from 0.93 to 1.06, and the probability of obtaining a LOD over 1.0 for an unlinked marker ranged from <0.001 to 0.05.

**Table 5:** Families showing slight evidence for linkage of myopia (Model 1) to chromosome 12q or 18p

AMISH FAMILIES					
FAMILY ID	TWO-POINT LOD		MULTIPOINT		
		
	Marker	Zmax	LOD	NPL	P value
3061	D12S1706	1.04	1.04	*	N/A
	D12S346	1.04	1.04	*	N/A
	D12S78	1.04	1.04	*	N/A
3049	D18S474	1.14	1.27	2.19	0.02
3053	D18S474	1.30	1.30	-*	N/A
3064	D18S63	1.30	1.30	-*	N/A

JEWISH FAMILIES					

FAMILY ID	TWO-POINT LOD		MULTIPOINT		
		
	Marker	Zmax	LOD	NPL	P value
20	D12S79	1.12	1.12	3.45	0.02
	D12S86	1.12	1.12	3.45	0.02
58	D12S1706	1.16	1.16	3.01	0.06
	D12S346	1.16	1.16	3.01	0.06
78	D12S85	1.07	1.07	3.38	0.01

* Only single affected parent-offspring pairs were genotyped in these families so the NPL analysis was uninformative in these families. However, the parametric LOD score analysis that uses both affected and unaffected family members was informative for linkage.

## Discussion

The overall results of these preliminary studies do not indicate any strong evidence for linkage of myopia in these families to the candidate regions on chromosomes 12 or 18. Although some families show marginal evidence of linkage to one of these regions, the results could be due to chance. Our negative results for these candidate regions have several possible explanations. First, the diagnostic criteria used in the previous studies [9,10] that implicated these candidate regions were based on limiting the affection status to the sphere component of a plus cylinder refraction. An individual was considered affected with high myopia if the sphere was equal to or greater than -6D regardless of the astigmatic error. Our study required an individual to have -1D in each meridian to be considered affected. Therefore, the criterion for being affected was quite different between the two studies. Second, the study population in our study included moderate and low myopes in addition to a small number of high myopes. None of our families showed strong aggregation of high myopia. Therefore, there were no families in our study recruited exclusively for high myopia and no families that would have been highly powerful for the detection of linkage to a high myopia trait. We utilized this study design to search for allelic heterogeneity with regard to the 18p and 12q loci thinking that one or both loci may predispose to moderate/mild forms of myopia. Thus, the current linkage analysis was done to test the hypothesis that other alleles at the candidate high myopia loci on chromosomes 18 and 12 might contribute to the etiology of moderate/mild myopia. The mild evidence of linkage in a few families indicates that this hypothesis cannot be fully ruled out for a very small proportion of families with mild forms of myopia. However, there is no strong evidence in favor of this hypothesis and strong negative evidence against linkage in most of the families in this study.

Previous studies attempting to confirm the high myopia loci on chromosomes 18 and 12 have yielded inconsistent results. Naiglin et al.[19]collected 23 French families with high myopia (spherical equivalent ≥ -6D) and performed a genome scan with 400 markers. Significant linkage was not found on 18p and 12q. Lam et al.[20] mapped 15 families with high myopia, ≥ -6.0D, using only 18p markers. Statistically significant (LOD > 3) linkage was not demonstrated although a multipoint LOD over 2.0 was observed, thus giving evidence of confirmation of the 18p candidate region. Mutti et al.[21] collected 53 families with varying degrees of myopia (affected ≥ -0.75D in each meridian) and genotyped the family members with 18p and 12q loci markers implicated in high myopia. No evidence of linkage to milder forms of myopia was found to the chromosome 18p and 12q loci previously associated with high myopia. Our study, although consistent with the results of Mutti et al.[21] was significantly different in design. First, Mutti et al. used a heterogeneous population that could decrease the chances of obtaining significant linkage for a minor gene effect from 18p or 12q if substantial ethnic heterogeneity exists. Both the Amish and Ashkenazi populations used in our study are more homogeneous and each sample was analyzed using marker allele frequencies estimated from the sample. Second, their study utilized 221 samples while we genotyped 613 individuals. Our power simulations predicted higher power in the presence of heterogeneity in our Ashkenazi families than was predicted for the Mutti et al. study. Our Amish families were of similar size and structure and so should have similar predicted power as the Ashkenazi families, and our combined analyses of the two data sets should provide much more power than that predicted by the simulations of the Ashkenazi families alone. The combination of the Mutti et al. study with the results presented here strongly suggest that these two candidate regions do not play a large role in the causation of moderate/mild myopia in several populations examined.

These studies suggest that myopia is complex and probably caused by the interaction of multiple genes with the environment. Therefore, to understand myopia it is necessary to apply the equation: Genes + Environment=Outcome. The difficulty here is the uncertainty surrounding both terms in the equation; ideally, one set of genetic factors will interact with one set of environmental influences to produce identical outcomes, but it is unknown whether this is always going to be the case. Therefore, to lessen the problem of multiple gene interaction as well as gene-environment interaction confounding the results, strategies to limit this problem should be utilized in the genetic mapping of myopia. The use of isolated populations is one approach to limiting the heterogeneity across populations and is the approach we are using for a genome wide scan in these families. Furthermore, the definition of myopia needs to be standardized so comparisons across studies can be made accurately. Previous studies have utilized different requirements with regard to affection status making cross comparisons difficult. In conclusion, we find little evidence implicating previously described susceptibility loci for high myopia on chromosomes 12 and 18 as being important in the etiology of common, moderate/mild myopia in our two population samples.

## Competing interests

None declared.

## Authors' contributions

Dwight Stambolian, Lauren Reider, Debra Dana, Robert Owens, and Elise Ciner recruited patients for the study. Melissa Schlifka carried out the genotyping in chromosomes 18 and 12. Dwight Stambolian and Joan E. Bailey-Wilson performed the study design and wrote part of the manuscript. Joan Bailey-Wilson oversaw all statistical analyses; Grace Ibay performed statistical analyses and wrote part of the manuscript; Taura Holmes, Betty Doan and Jennifer O'Neill assisted with analyses of the data. All authors read and approved of the final manuscript.

## Pre-publication history

The pre-publication history for this paper can be accessed here:


